# Xanthurenic Acid Binds to Neuronal G-Protein-Coupled Receptors That Secondarily Activate Cationic Channels in the Cell Line NCB-20

**DOI:** 10.1371/journal.pone.0048553

**Published:** 2012-11-06

**Authors:** Omar Taleb, Mohammed Maammar, Daniel Brumaru, Jean-Jacques Bourguignon, Martine Schmitt, Christian Klein, Véronique Kemmel, Michel Maitre, Ayikoe Guy Mensah-Nyagan

**Affiliations:** 1 Biopathologie de la Myéline, Neuroprotection et Stratégies Thérapeutiques, UMR_S INSERM U-1119, Université de Strasbourg, Faculté de Médecine, Strasbourg, France; 2 Laboratoire d’innovation Thérapeutique, CNRS UMR-7200, Université de Strasbourg, Faculté de Pharmacie, Illkirch, France; University of New South Wales, Australia

## Abstract

Xanthurenic acid (XA) is a metabolite of the tryptophan oxidation pathway through kynurenine and 3-hydroxykynurenine. XA was until now considered as a detoxification compound and dead-end product reducing accumulation of reactive radical species. Apart from a specific role for XA in the signaling cascade resulting in gamete maturation in mosquitoes, nothing was known about its functions in other species including mammals. Based upon XA distribution, transport, accumulation and release in the rat brain, we have recently suggested that XA may potentially be involved in neurotransmission/neuromodulation, assuming that neurons presumably express specific XA receptors. Recently, it has been shown that XA could act as a positive allosteric ligand for class II metabotropic glutamate receptors. This finding reinforces the proposed signaling role of XA in brain. Our present results provide several lines of evidence in favor of the existence of specific receptors for XA in the brain. First, binding experiments combined with autoradiography and time-course analysis led to the characterization of XA binding sites in the rat brain. Second, specific kinetic and pharmacological properties exhibited by these binding sites are in favor of G-protein-coupled receptors (GPCR). Finally, in patch-clamp and calcium imaging experiments using NCB-20 cells that do not express glutamate-induced calcium signals, XA elicited specific responses involving activation of cationic channels and increases in intracellular Ca^2+^ concentration. Altogether, these results suggest that XA, acting through a GPCR-induced cationic channel modulatory mechanism, may exert excitatory functions in various brain neuronal pathways.

## Introduction

In the brain, tryptophan is the precursor of many neuroactive compounds generated via different biochemical pathways [1]. The major part of tryptophan degradation is through oxidation via the kynurenine route that leads to the production of several neuroactive intermediates, either neurotoxic or neuroprotective [2]. XA comes from the transamination of 3-hydroxykynurenine formed from kynurenine by a specific hydroxylase, while kynurenic acid is the direct transamination product of kynurenine. Thus, XA and kynurenic acid are closely structurally related but possess very different biological roles. However the properties of XA in the brain remain elusive. Kynurenic acid modulates NMDA and α7 nicotinic receptor activities and possesses inhibitory properties, while other kynurenine pathway intermediates like quinolinic acid is considered to be involved in brain inflammatory diseases via its neurotoxic effects [3]–[5]. Two compounds, 3-hydroxykynurenine and 3-hydroxyanthranilic acid, are considered as free radical generators, and the deleterious accumulation of the former substance is thought to be reduced by its transformation into XA [6]. Apart from its involvement in detoxification processes, no specific or neuromodulatory function has until now been identified for XA in the brain. It is present in blood and urine at concentrations of 0.7 and 5–10 µM respectively, but is also heterogeneously distributed in brain tissue at an average concentration of 1 µM [7]. This XA concentration can be modified by peripheral administration of this substance that apparently penetrates the brain freely [7], [8].

Up to now, only four different types of information are available concerning XA roles in various experimental models. First, it has been reported that XA possesses some toxicity in senile cataract and certain infectious diseases, and leads to apoptotic-like cell death in various cell cultures at a concentration of 10 µM [9], [10]. Second, XA seems to trigger gametogenesis of the malaria parasite in mosquito blood via a mechanism that involves cGMP-dependent protein kinase and intracellular Ca^2+^ increases [11]. Third, kynurenine metabolites including possibly XA, have been implicated in the pathophysiology of several neurodegenerative disorders, where inflammatory and aberrant immunological processes are observed [12]. Finally, the presence of XA in the mammalian brain, its distribution, transport and release have led to the idea that this substance could be involved in some signaling pathways, adding XA to the list of neuroactive substances derived from the kynurenine pathway [7]. Recently, XA has been identified as a selective endogenous Group II (mGlu 2 and 3) metabotropic glutamate receptor ligand in vitro and has been found to mimic the in vivo effects on sensory inhibition in the thalamus afforded by other Group II mGlu receptor agonists [13]. The present study generated original and key results showing that XA binds to brain receptors whose kinetics, distribution and pharmacology are specific. When stimulated by XA or synthetic related ligands, differentiated neuroblastoma cells exhibited an excitatory response that led to cytosolic calcium ion increase. Altogether, the data described herein strongly support a neurotransmitter/neuromodulator role for XA in the brain.

## Materials and Methods

Xanthurenic acid, kynurenic acid, L-kynurenine, 3-OH-DL-kynurenine, 5-OH-tryptophan, picolinic acid and 3-OH-anthranilic acid were purchased from Sigma-Aldrich (Saint-Quentin, France). [^3^H]-XA was obtained from Amersham (Cardiff, UK; 38 Ci/mmole). Radiochemical purity (97.9%) was evaluated by HPLC with a gradient of 0.1% trifluoroacetic acid in water/0.1% trifluoroacetic acid in acetonitrile on a Betabasic C18 column (particle size 5 µm). The material co-chromatographs with commercially available material in the above chromatographic system and the mass spectra were consistent with the proposed structure and a non-labeled reference (Amersham specifications).

### Synthesis of Xanthurenic Analogues

4-hydroxyquinoline-2-carboxylic acid analogues (NCS-482, XT-21 and NCS-486) were synthesized in the laboratory of Dr J.J. Bourguignon. Cyclocondensation of commercially available ortho-methoxy anilines 1a,b (Figure S1) with dimethyl acetylene dicarboxylate followed by cyclization under thermal condition furnished the substituted ortho–methoxy quinolines 2a, b (Figure S1) as described in the literature [14]. The 6-bromo derivative (2b) was submitted to a palladium–catalyzed reaction with phenylboronic acid to give 2c (Figure S1) after purification on flash chromatography [15]. Deprotection of the methoxy group was achieved with BBr_3_. Finally carboxylic acid 3a and 3b (NCS-482 and XT-21, respectively; Figure S1) were obtained after hydrolysis of the ester group in a solution of potassium iodide in 95% phosphoric acid [16].

NCS-486 was prepared in a two step reaction starting from readily available methyl-8-nitro-4-oxo 1,4 dihydroquinoline 2-carboxylate [17]. Reduction of the nitro group was accomplished by a catalytic hydrogenation. Hydrolysis of the methyl ester was performed as previously described [16]. The structures of XA analogues were confirmed by NMR analysis.

### Animals

Adult male albino Wistar rats weighing 350 to 400 g, bred in the Faculty of Medicine (Strasbourg, France) were used for experiments. The rats were housed in individual plastic cages (40 cm · 25 cm · 25 cm) on a standard 7∶00 to 19∶00 h light/dark cycle with free access to food and water. Procedures involving animals and their care were conducted in compliance with a European Communities Council Directive (86/609/EEC) and under the supervision of authorized investigators. In addition, all of the protocols were reviewed and approved by the Alsace Head Office of the French Department of Veterinary and Public Health Guide for the Care and Use of Laboratory Animals with the agreement number 67–186.

### Crude Synaptosomal Membrane Fraction from Rat Brain

Brains were rapidly extracted from adult male Wistar rats killed by decapitation after being stunned. All the following procedures were done with buffers cooled at 0°C. Brains were homogenized with a motor driven Teflon/glass apparatus in 10 vol (W/V) of sucrose 0.32 M containing potassium phosphate buffer pH 7.2 and 2 mM EDTA. After centrifugation at 1,500 g for 10 min to remove the crude nuclear fraction, the supernatant was spun at 35,000 g for 20 min. The pellet obtained was resuspended in lysis buffer (2 mM EDTA/water pH 6.0 at 0°C, spun at 100,000 g for 20 min). After washing the pellet with potassium phosphate buffer 50 mM, pH 6.0, the crude membrane fraction was directly used for further experiments or stored at −80°C.

### Cell Culture Preparations

NCB-20 cells are a hybrid between mouse neuroblastoma N18TG2 and Chinese hamster embryonic day 18 brain cells, which express many properties characteristic of neurons [18]. The cell line was obtained from the IGBMC (Institute of Genetic and Cellular and Molecular Biology, Strasbourg) cell collection. These cells were cultured essentially as described previously [19], [20]. Briefly, cells were plated (30,000 cells/ml) in 35-mm Petri dishes. Each dish contained 2 ml of Dulbecco’s modified Eagle medium supplemented with 10% fetal calf serum, penicillin (50 U/ml), streptomycin (50 µg/ml) and 1 mM dibutyryl adenosine 3′, 5′-cyclic monophosphate. Cultures were incubated in a humidified CO_2_ (5%) incubator.

Human neuroblastoma cells IMR-32 (ATCC number: CCL-127) were cultured in minimum essential medium Eagle supplemented with 2 mM L-glutamine and Earle’s BSS adjusted to contain 1.5 g/l sodium bicarbonate, 0.1 mM non-essential amino acids, 1.0 mM sodium pyruvate and 10% fetal calf serum. Cultures were maintained for one week at 37°C in the presence of CO_2_ (5%).

### Crude Membrane Fractions from Neurons in Culture

Neurons were scraped into potassium phosphate buffer 50 mM, EDTA 2 mM, pH 6.0 at 0°C. After centrifugation at 2,000 g for 3 min to eliminate nuclei and large fragments, the supernatant was spun down at 15,000 g for 10 min; the pellet was washed with the same buffer and directly used for binding experiments.

### Binding Experiments

Membranes (from brain or cell cultures) were incubated for 30 min at 0°C in 50 mM Pipes buffer, pH 7.4 in the presence of [^3^H]-XA (2 µCi per µmole total XA at various concentrations). Non-specific binding was determined with 1 mM non-radioactive XA in the medium. Bound from free ligand was separated by rapid filtration on 25 mm Whatman GF/B filters and washing with incubation buffer (3×3 ml at 0°C). Radioactivity on filters was measured by liquid scintillation counting.

### Distribution of XA Binding on Rat Brain Slices

Once the optimal conditions for binding were ascertained and preliminary binding kinetics determined, 3 male Wistar rats were killed by decapitation, the brains rapidly removed, fixed for 60 sec in −40°C isopentane on dry ice, and brought to −20°C for 1 h before cutting. The brains were cut into 20 µm sections at −20°C and then thaw-mounted onto gelatin-coated coverslips. These were stored at −80°C until autoradiography was performed. Based on the preliminary binding experiments, the following conditions were used for [^3^H]-XA autoradiography. Slides were preincubated for 15 min at 20°C in 50 mM, pH 7.4 potassium phosphate buffer, then incubated for 30 min at 4°C in the same buffer with 3 µM [^3^H]-XA (38 Ci/mmol). Non-specific binding was determined in the presence of 1 mM non-radioactive XA in the incubation medium. The slides were washed twice for 10 sec in ice-cold buffer, dipped in deionized water, dried in a stream of cold air, and opposed to tritium-sensitive film (Amersham) which was then placed in X-ray cassettes with appropriate [^3^H] microscale standards, exposed for 4–7 days, developed, and analyzed by computer assisted densitometry.

### XA-induced [^35^S]GTP-γ-S Binding

The Scintillation Proximity Assay (SPA) of Amersham was used. [^35^S]GTP-γ-S binding to rat brain membranes was determined in the presence of 0.5 to 50 µM of XA. Preliminary experiments have shown that optimum [^35^S]GTP-γ-S binding stimulated by XA was obtained with 5 µM GDP and 20 mM MgCl_2_. Brain synaptosomal membranes were thus incubated in assay buffer (pH 7.4) containing 20 mM HEPES, 100 mM NaCl, 20 mM MgCl_2_ with 0.3 nM [^35^S]GTP-γ-S and 5 µM GDP for 30 min at room temperature. Non-specific binding was determined in the presence of 200 µM GTP. WGA SPA beads were added and the mixture was incubated at room temperature for a further 30 min. The plates were centrifuged at 1,200 g for 5 min and SPA cpm were determined by scintillation counting.

### Electrophysiological Recordings

NCB-20 cells were plated at low density (10^4^ cells/dish) in 35-mm dishes and differentiated with 1 mM db-cAMP. The culture medium was replaced twice a week and the cells were used after 5 days in culture. Cells were recorded either in cell-attached or whole-cell configuration of the patch-clamp technique [21] using an Axopatch-B200 amplifier (Axon Instruments, CA, USA) in its voltage-clamp mode. Current signal was low-pass filtered at 1 kHz before a 2 kHz digitization using the Digidata 1322A card interface (Axon Instruments, CA, USA) and Pclamp software (Axon instruments, CA, USA). Junction potentials were calculated using the routine procedure in the Clampex software (Axon instruments, CA, USA) and taken into account for current-voltage (I–V) relationships and reversal potential calculation according to the recording configuration.

#### Whole-cell recording

Tight-seals were performed with pulled pipettes from borosilicate capillaries whose tip resistance was 2.2±0.2 MΩ (n = 20) when filled with high chloride containing medium (in mM: KCl 125, MgCl_2_ 2, EGTA/K 5.5, CaCl_2_ 1, HEPES 10, pH = 7.2 with KOH). In some experiments, we also used a low chloride pipette medium of the following composition (in mM): K-gluconate 122, KCl 3, NaCl 5, MgCl_2_ 2; EGTA-K 5.5, CaCl_2_ 1, HEPES 10, pH = 7.2 with KOH. In the latter case, pipette resistance was 4.1±0.1 MΩ (n = 56). The mean cell membrane capacitance value obtained was 53.4±6.3 µF (n = 132 cells). Isolated NCB-20 cells were selected for recording and were continuously superfused with a control solution containing: (mM) NaCl 135, KCl 5, MgCl_2_ 2, CaCl_2_ 0.5, HEPES 10, D-glucose 10, pH adjusted to 7.4 with NaOH. Under these conditions, the calculated junction potential had a value of 5.2 and 14.4 mV when using high and low chloride-containing pipette medium respectively.

#### Cell-attached recording

For cell-attached recording the pipette was filled with a medium containing (in mM): N-Methyl-D-Glucamine (NMDG) chloride 140, KCl 1, MgCl2 10, EGTA 5, TEA-Cl 15, HEPES 10, pH = 7.4. To maintain the cell membrane potential close to zero, the recorded cells were continuously superfused with a bath solution containing (in mM): KCl 140, NaCl 1, MgCl_2_ 1, CaCl_2_ 0.5, EGTA-K 1, HEPES 10, D-glucose 10, pH = 7.4. The calculated junction potential in these conditions was −11.4 mV. Drugs were dissolved in DMSO and diluted in control solutions such that DMSO in the applied solution was diluted by a factor of at least 10^4^. Drugs were applied to the recorded cells through a multi-barrel perfusion system, the rapid solution exchanger RCS-160 (Bio-Logic, Grenoble, France). Each barrel had a 1 mm inner diameter and the selected tube was placed about 100 µm from the recorded cell. The first tube was filled with control solution and was used to maintain a continuous superfusion of the recorded cell between applications. The selected tube was placed in front of the cell by axial rotation of calibrated angles and the application started when the selected tube takes place in front of the cell. The parameters of the solution exchanger were set so as a complete solution exchange around the recorded cell was achieved in about 2 s.

Data were analyzed offline using the clampfit routine of the Pclamp software package. The given results are means±SEM and statistical significance of the difference between means was determined using one way ANOVA followed by Dunnett's Multiple Comparison test.

### Monitoring of [Ca^2+^]_i_ Changes

The method of cellular calcium measurement used was as described previously [22]. Briefly, NCB-20 cells were plated in glass-bottomed culture dishes and incubated at 37°C for four days. Cells were loaded with the fluorochrome by incubation in Krebs medium (in mM: NaCl 145, KCl 2.7, MgCl_2_ 1, CaCl_2_ 1.8, D-glucose 10, HEPES 10, pH 7.4) containing 10 µM fluo-4 AM and 0.02% pluronic acid F127 for 30 min at 37°C in the dark. Then the cells were washed with Krebs for 30 min at room temperature in the dark. All settings of the laser (Argon-Krypton laser, turned to 488 nm), optical filter (BF 530/30) and microscope (Leica TCS-SP confocal inverted microscope) as well as data acquisition were controlled by the LCS (Leica Confocal System) software. Images were taken using a 256 gray scale with a photomultiplier and the “glowoverglowunder” system (Leica) using a 40× objective, NA 1.2, with an electronic zoom of 2 to 4 fold. The image size was 512×512 pixels and before each measurement, serial sections were acquired in the vertical axis to choose the equatorial section of the cells.

Images were recorded at a frequency of 0.1 Hz. Analysis was performed by defining regions of interest (ROI) in the first image. Values are expressed as the percentage of fluorescence change with respect to control and calculated as *F*/*F*_0_ *(%)* (where *F* is the fluorescence intensity at a given time and *F*_0_ the basal fluorescence intensity at the beginning of the experiment).

The cells were continuously superfused with normal Krebs medium and transiently submitted to the tested drug using a peristaltic pump with a flow rate of 1 ml/min. For each analyzed cell, the mean and the standard deviation (SD) of the *F*/*F*_0_ of fluorescence were measured during 1–2 min before (base line) and after (signal) drug application and compared. Statistical significance of the difference between means was determined using one way ANOVA followed by Dunnett's Multiple Comparison Test for repeated measurements on absolute levels of fluo-4 fluorescence [22].

## Results

### Binding Sites for XA on Brain Synaptosomal Membranes and on Neuronal Cell Lines

Saturation experiments performed with increasing concentrations of radioactive XA showed the presence of binding site(s) for this compound on crude synaptic membranes isolated from rat brain. Non-specific binding was linear and specific binding was proportional to protein concentration under our experimental conditions (data not shown). Optimum pH for specific binding was about 7.5±0.3 as determined in Pipes buffer from pH 5.5 to 8.0 (data not shown).

**Figure 1:**
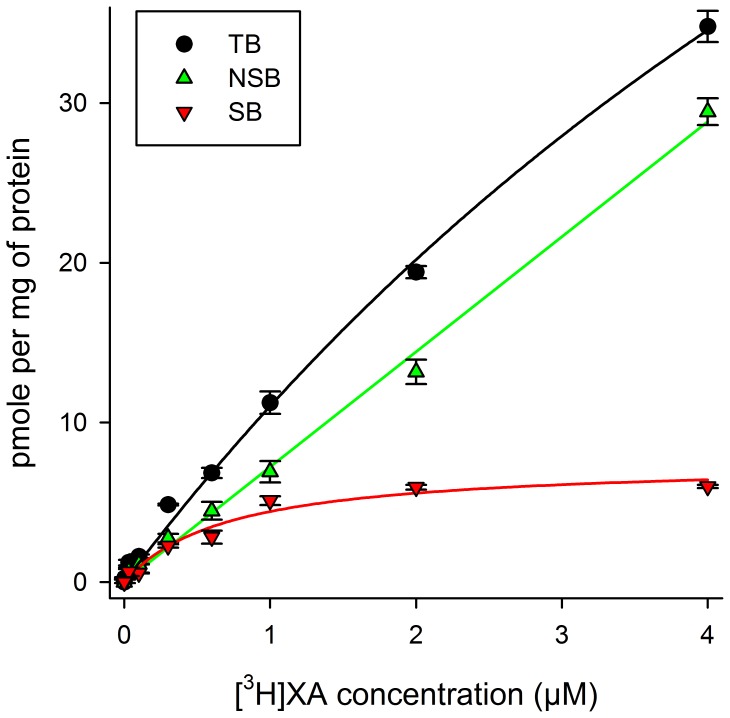
Saturation curve showing specific binding of [^3^H]XA displaced by 1 mM non-radioactive XA on crude synaptosomal membranes from rat brain. TB: total binding; NSB: non-specific binding; SB: specific binding. Non-linear regression lines with GraphPad Prism Program; R^2^ = 0.78. Each point is the mean±SD of three experiments made in triplicate at each concentration. Kd = 0.74 µM; Bmax = 7.5 pmoles/mg protein.

For crude brain synaptosomal membranes, IMR-32 and NCB-20 neuroblastoma cells, dissociation constant (Kd) values of respectively 0.74, 0.75 (Bmax 5.2±0.4 pmoles/mg protein) and 1.30 µM (Bmax 4.8±0.5 pmoles/mg protein) were obtained (non linear regression lines with the GraphPad/Prism software, R^2^ = 0.96; 0.71 and 0.90 respectively). For brain membranes (Figure 1), a calculated Bmax value of 7.5±0.4 pmoles/mg protein was obtained and association (K_on_) and dissociation (K_off_) rate constants were determined (pH 7.4 and 0°C). K_on_ was calculated according to the following relation: K_on_ = (K_ob_-K_off_) [L] (GraphPad Prism Program, San Diego, CA). K_ob_ was obtained by monitoring the amount of [^3^H]-XA (0.2 µM; 2 µCi/µmole) specifically bound with time (30 sec to 40 min; 3 experimental points every minute). Under these conditions, the binding began to saturate after 3 min (goodness of fits, R^2^ = 0.75) and K_ob_ was 1.44 min^−1^ (GraphPad/Prism program). K_off_ was determined under the same conditions in the presence of 1 mM non-radioactive XA and showed a value of 1.26 min^−1^ (R^2^ = 0.74). From these results, the calculated Kd was 1.4 µM which is close to those obtained using saturation experiments.

### Pharmacological Properties of XA Binding Sites

**Figure 2:**
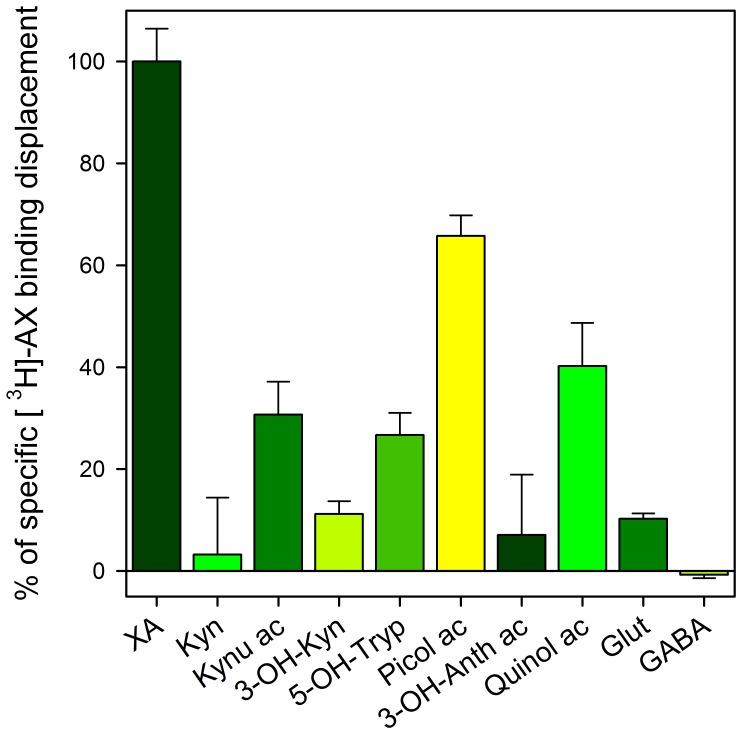
Effects of amino acid neurotransmitters, selected XA-related analogues and compounds of the kynurenine pathway on XA binding sites. Each compound was tested at 200 µM concentration in the presence of radioactive XA. Displacement of radioactive XA induced by 200 µM XA was arbitrarily set at 100%. Result values are the mean of 3 experiments performed in triplicate±SEM.

Two approaches were used for screening the pharmacological properties of XA specific binding. First, we tested at rather high concentration (200 µM) a battery of putative ligands for these sites. Among these, glutamate and GABA exhibited very low potency (Figure 2). The fact that glutamate displaced not more than 10% of radioactive XA eliminates possible binding of XA on glutamate receptors under our conditions [13].

Among XA related compounds that were tested for their ability to displace XA specific binding (Figure 2), kynurenic acid was a weak competitor (30% displacement at 200 µM concentration), picolinic and quinolinic acids had much more significant effects (60% and 40% displacement respectively). For the more powerful competitor picolinic acid, a dose-dependent displacement of [^3^H]XA binding was performed (data not shown) and a mean IC_50_ value of 41±5 µM was obtained, indicating its low affinity for the XA binding sites. Therefore, this screening test revealed the specificity of XA among the compounds tested.

Secondly, to extend the pharmacological profile of XA, this compound was tested on high-throughput profile screening which involved a collection of 75 transmembrane and soluble receptors, ion channels and monoamine transporters (Cerep, France Laboratories, Poitiers). XA was tested on these collections of proteins at 10 µM concentration. No significant competitive properties of XA were observed on the binding of specific ligands for each of these proteins (for detailed information on the protein collection, see the CEREP catalogue at www.cerep.fr/cerep/users. In particular, XA was found to have no effect on adenosine, benzodiazepine, dopamine, GABA, canabinoid, histamine, muscarinic, opioid, purinergic and serotonin receptors.

### Autoradiographic Distribution of XA Binding Sites in the Rat Brain

**Figure 3:**
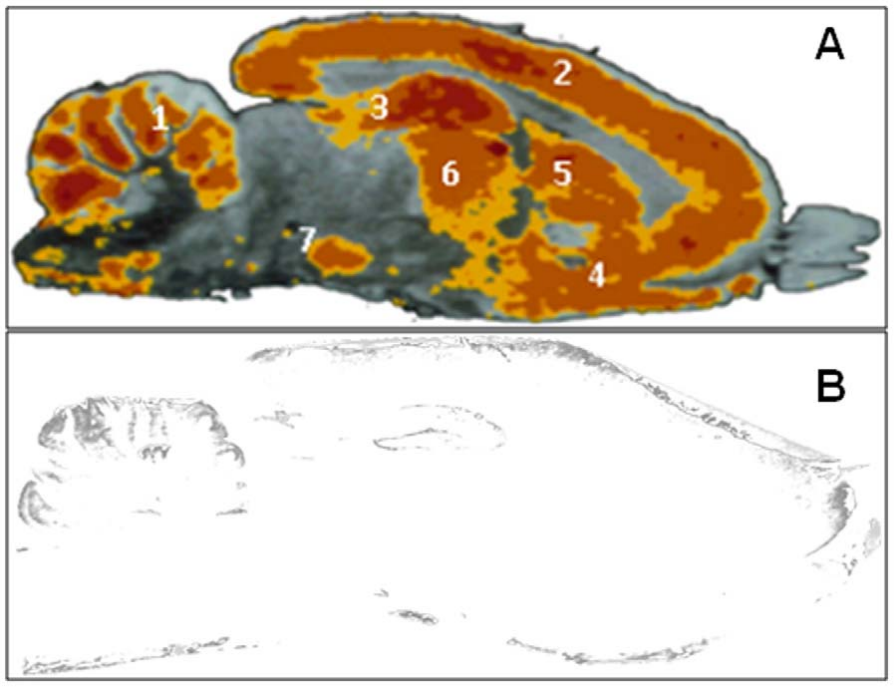
Distribution of rat brain XA binding sites. Autoradiogram of sagittal sections of rat brain (lateral 1.90 mm, according to Paxinos and Watson (1998)), showing in **A** the distribution of XA binding sites as a pseudo-color image (dark brown/red: +++; brown: ++; yellow: +). 1: cerebellum; 2: cortex; 3: hippocampus; 4: olfactory tracts; 5: striatum; 6: thalamus; 7: mesencephalic dopaminergic nuclei (A_9_/A_10_). In the presence of an excess of non radioactive XA (1 mM), a very faint image can be detected on autoradiographies (**B**).

Reversible high affinity XA binding (Figure 3 and Table 1) appeared to be especially localized in the rostral part of the rat brain, including diencephalic and telencephalic structures (cortex, hippocampus, striatum, thalamus, olfactory tracts). By contrast, the mesencephalic region was almost completely devoid of binding sites, with the exception of dopaminergic mesencephalic nuclei (A_9_/A_10_), but this result is not absolute considering the definition levels of the image analysis. In the caudal part of the brain, there was also a low density of sites with the notable exception of cerebellum (Figure 3 and Table 1). These results highlight the heterogeneous distribution of XA binding sites in the rat brain, the richest regions (dorsal hippocampus and caudate nucleus) expressing about 250 fmoles of sites per mg tissue while some other regions did not display detectable binding.

**Table 1:** Quantitative autoradiographic densities of binding sites in brain regions.

Brain regions	Density of sites (fmoles/mg equivalent tissue)	Relative percentage (%)
Dorsal hippocampus	268.5±5.3	100
Caudate nucleus lat. part	252.0±4.0	93
Central amygdaloïd nucleus	249.4±5.6	92
Accumbens nucleus lat. part	247.3±12.8	91
Pyriform cortex	233.5±11.9	86
Parietal cortex	228.2±3.0	84
Cerebellar lobules	226.0±7.6	83
Post. medial thalamic nucleus	220.9±9.0	81
Dorsomedial hypothalamus	220.7±9.5	81
Cingulate cortex ant. part	219.7±6.4	81
Prefrontal cortex	219.7±14.0	81
Olfactory tracts	221.2±10.6	81
Lateral septal nucleus	208.9±7.2	76
Mediodorsal thalamic nucleus	203.7±5.1	75
Globus pallidus	183.5±8.7	67
Medulla oblongata	162.2±6.6	59
Occipital cortex	160.0±37.8	59
Medial septal nucleus	149.4±15.1	55
Ventral hippocampus	110.0±2.2	40
Interpeduncular nucleus	106.8±2.2	39
Substantia nigra (A_9_)	104.6±1.8	38
Dorsal raphe nucleus (B_7_)	94.1±7.7	34
Periacqueductal gray matter	94.1±5.2	34
Ventral tegmental area (A_10_)	89.8±5.6	32
Temporal cortex	82.4±6.8	30
Median raphe nucleus (B_8_)	70.6±2.2	25

Results are mean±SD of the quantitative optical density in the autoradiographic trace (Biocom program) by reference to standard autoradiographic scales (Amersham). Results are mean of 8 to 10 measures in each brain region.

The limit of the signal detection is about 50 fmoles/mg equivalent tissue.

### XA Stimulates [^35^S]-GTP-γ-S Binding on Rat Brain Synaptosomal Membranes

**Figure 4:**
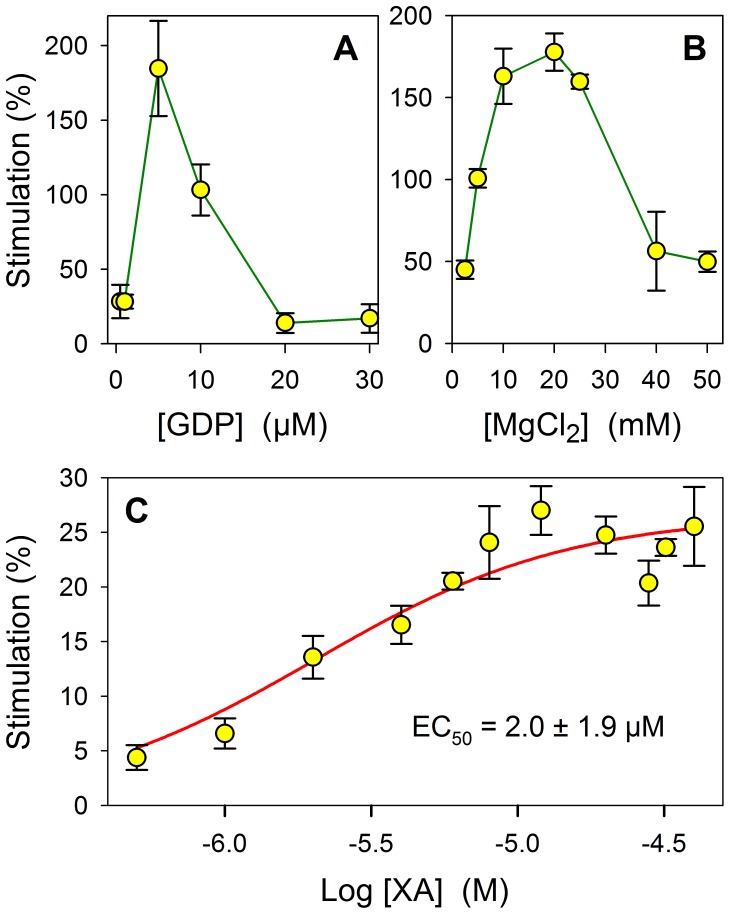
XA-induced GTP-γ-S binding characteristics. A and **B** show the optimal concentration level of GDP and MgCl_2_ respectively that induce maximal GTP-γ-S binding in the presence of a fixed concentration of XA (5 µM). **C**: EC_50_ of GTP-γ-S binding to crude synaptosomal membranes from rat brain in the presence of increased XA concentrations from 0.5 to 50 µM. Non-linear fitting using the GraphPad Prism program, each experimental point was the mean±SEM of three results obtained in triplicate at each concentration.

Preliminary experiments showed that XA-induced radioactive GTP binding to rat brain synaptosomal membranes occurred optimally for GDP and MgCl_2_ concentrations of 5 µM and 20 mM respectively (Figures 4A and 4B). Using XA concentrations ranging from 0.5 to 50 µM, GTP binds to the membrane fraction with an EC_50_ of 2.0±1.9 µM (Figure 4C).

### Electrophysiological Responses Induced by XA in NCB-20 Cells

Our aim in these experiments was to describe a XA cellular effect mediated by specific receptors differing from that reported through class II metabotropic glutamate receptors (mGluR2/3) [13]. The best way for this initial characterization of XA cellular effects was to use a neuronal preparation that does not express these mGluR receptor types. Differentiated NCB-20 cells were chosen because they express a neuronal phenotype [18], do not express glutamate receptors that increase cellular calcium levels [23] and exhibit XA binding sites. However, application of this cellular model is limited to this first identification of XA neuroactivity, and the active effect of XA should be confirmed on neurons of different species.

#### Whole-cell recording of the XA response

XA responses were recorded from db-cAMP differentiated NCB-20 cells after 5 days of culture. An XA (1 to 30 µM) induced response was frequent and present in about 71% of the cells tested.

**Figure 5:**
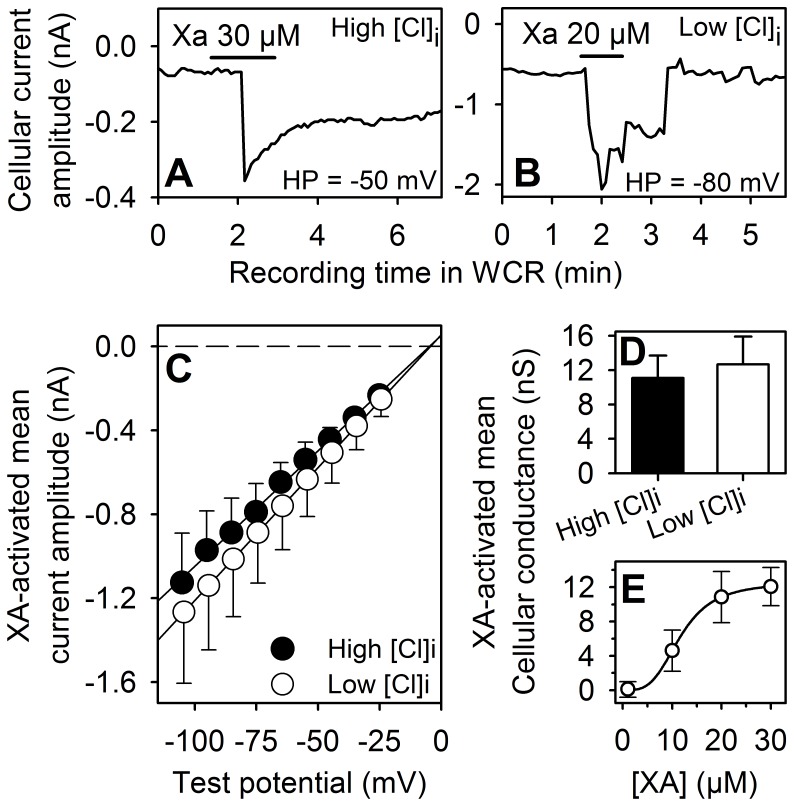
Whole cell recording of XA responses from differentiated NCB-20 cells. **A** and **B**: Current traces recorded in conditions of high (A) or low (B) cytoplasmic chloride ([Cl]i) concentrations. XA was applied to the recorded cells as indicated by the horizontal bars. **C**: Mean I–V relationships obtained in conditions of low (filled circles) or high (open circles) [Cl]i (5 cells in each condition). The dashed and continuous lines are the zero current level and linear regression to the data points respectively. Note that the reversal potential of XA responses did not change (−4.8 and −4.4 mV) with cellular chloride activities and was close to the cationic reversal potential which had a value of −0.9 and 0.4 mV in conditions of high and low cellular Cl concentrations respectively. **D**: mean cellular conductances activated by XA (20–30 µM) in high (black bar) and low (white bar) Cl concentrations respectively (mean±SEM, n = 7 cells). **E**: Dose-response relationship of XA. The continuous curve is the optimized Hill equation to the data points. Optimization parameters were EC50 = 11.7 µM, maximal conductance = 12.5 nS and Hill coefficient = 3.5.

The XA response consisted of an inward current at negative membrane potentials (Figure 5A and 5B). Figure 5C represents I–V relationships of the mean peak current amplitude induced by XA applications in conditions of high or low chloride-containing pipette medium. In both conditions the mean peak current amplitude was linear in the potential range tested. Comparable cellular conductance (Figure 5D) and reversal potential mean values were obtained at high (3 cells) and low chloride (4 cells) conditions and corresponded to 11.1±2.6 and 12.7±3.2 nS and −3.3±4.0 and −3.8±2.5 mV respectively. These results suggest that the XA-induced current was carried by cationic ions. The XA activated conductance was dose-dependent (Figure 5E) with an EC_50_ of 11.7±2.2 µM.

#### Cell-attached recording of the XA response

**Figure 6:**
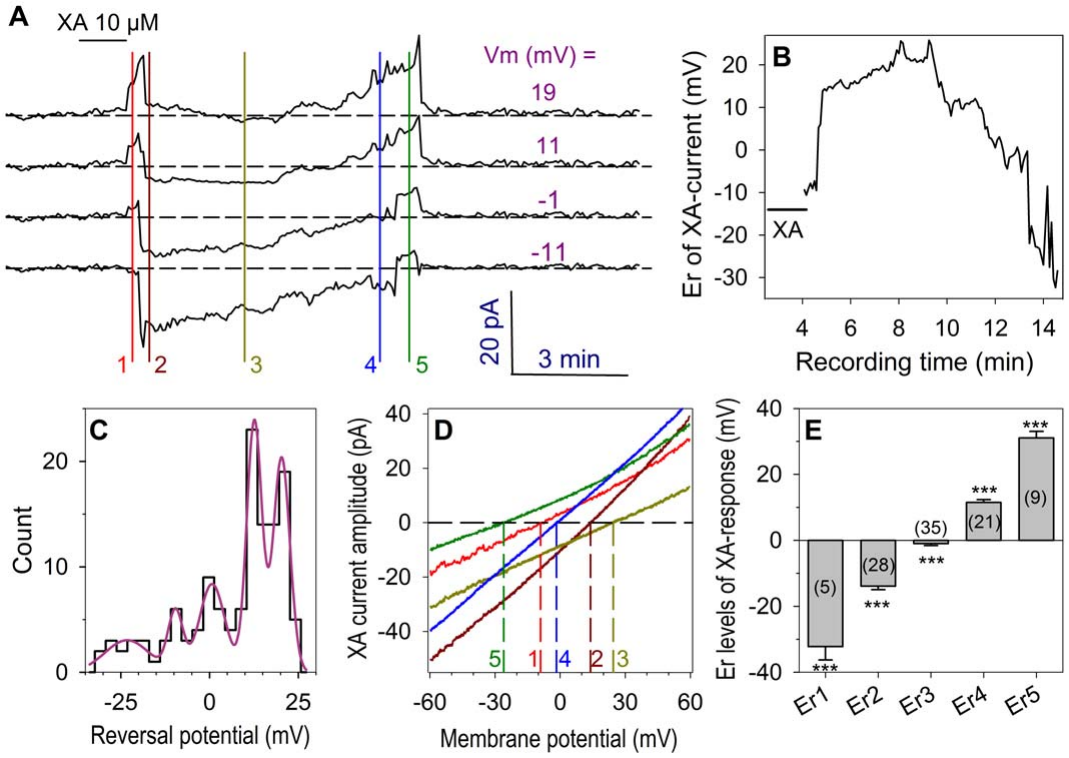
Reversal potential of XA-induced responses in the cell-attached configuration. The membrane patch was recorded in the NMDG-Cl condition and stimulated by a ramp potential protocol (−70 to 90 mV, 600 ms duration) at a frequency of 0.2 Hz. **A**: Membrane patch current traces recorded at different membrane potentials (Vm) as indicated in the figure. The colored vertical lines 1 to 5 indicate the different steps of the Er variations. The dashed lines represent the zero current level for each current trace. **B**: Time evolution of the Er value obtained by the ramp potential stimulation protocol during the XA response given in **A** (control current was subtracted). **C**: Frequency histogram of the Er values obtained during the XA response. The distribution was best fitted by the sum of 5 Gaussians with parameter values of: mean Er = −23.2, −9.6, 0.8, 12.6 and 20.3 mV, standard deviation σ = 6.3, 2.1, 3.6, 2.1 and 2.3 and amplitude = 3.0, 5.6, 8.4, 23.8 and 20.4 respectively. **D**: I–V relationship of the XA-induced current recorded at the times indicated by numbers 1 to 5 in **A**. The reversal potential for each I–V curve is indicated by dashed vertical lines (1 to 5). **E**: Mean data (± SEM) of the reversal potential classes (Er1 to Er5) obtained from the different XA responses. In parenthesis are given the number of time a given Er class was observed overall the XA responses. ***: p<0.001.

One approach to demonstrate the implication of a diffusible second messenger in G-protein responses is to record a response from a membrane-patch isolated from the rest of the cell membrane that is exposed to the agonist stimulation. This was done using the cell-attached configuration of the patch-clamp technique. The cells were bathed with a KCl solution (see methods) so as to clamp the membrane potential of the recorded cell close to zero mV. Figure 6A illustrates a membrane patch response induced by application of 10 µM XA to the recorded cell.

Unexpectedly, the cell-attached configuration using NMDG and Mg^2+^ as major monovalent and divalent cation ions respectively revealed a reversal potential (Er) for XA responses that could change across a large range of potentials during the same response. Thus, we used a voltage ramp protocol that allowed us to follow Er time evolution at a frequency of 0.2 Hz. In the case illustrated in figure 6A, the Er evolved sequentially (positions 1 to 5 in figure 6A) from an initial value of −9 mV to levels of about 14, 25, −1 and −26 mV (Figure 6B). I–V curves corresponding to each of these levels are given in figure 6D. These levels were also obtained from the frequency histogram (Figure 6C) of the reversal potential data given in figure 6B. This frequency distribution was best fitted with a sum of 5 Gaussians with mean parameter values of Er1 =  −23.2, Er2 =  −9.6, Er3 = 0.8, Er4 = 12.6 and Er5 = 20.3 mV. However, in the total XA-responses recorded in these conditions, the five Er levels were not always present together in a given XA response, but rather as a combination of a subset. Particularly, levels Er2, Er3 and Er4 were by far the most frequent. The global Er data mean values (Er1 =  −32.2±4.0, Er2 =  −13.9±1.1, Er3 =  −1.0±0.6, Er4 = 11.5±0.8 and Er5 = 31.1±2.0 mV) are illustrated in figure 6E. These results suggest that XA activated cationic channels of different selectivity toward K^+^, NMDG^+^ and Mg^2+^ ions.

#### Pharmacological properties of Xanthurenic responses using synthetic analogs

**Figure 7:**
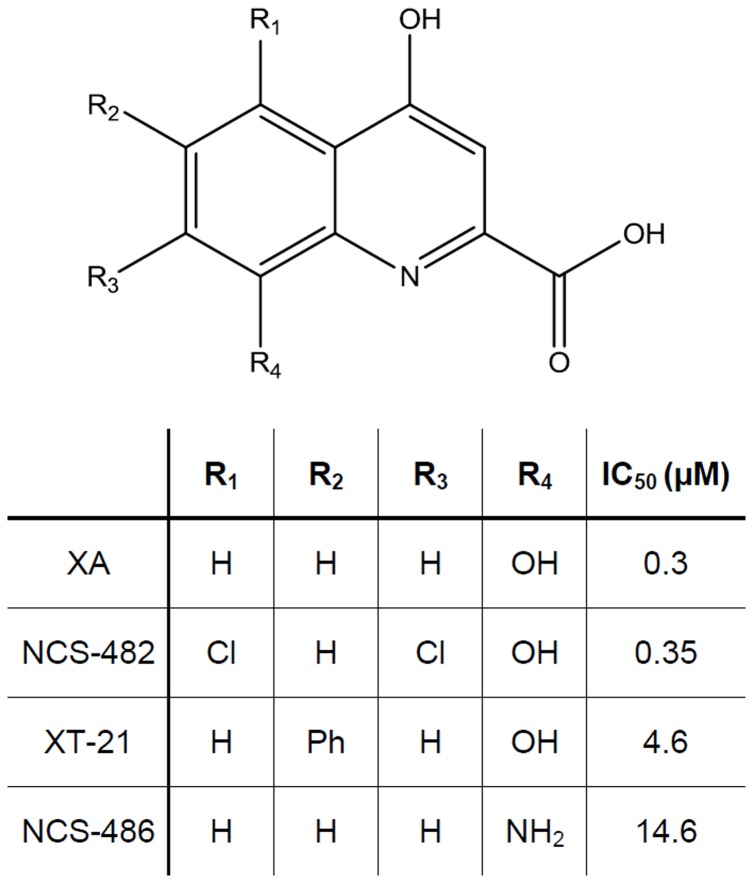
Structural representation of XA and selected analogs and their IC_50_ of [^3^H]XA binding displacement. XA analogs were selected for their agonist (NCS-482 and XT-21) or antagonist (NCS-486) properties.

Among the synthesized XA analogs, we selected some of them for their affinity for XA binding sites and tested their functional properties in patch-clamp experiments. Three molecules (NCS-486, NCS-482 and XT-21, Figure 7) were selected for their antagonistic (NCS-486) or agonistic properties (NCS-482 and XT-21). These three analogs displaced XA binding with an IC_50_ of 14, 0.35 and 4.6 µM respectively (Figure 7).

**Figure 8:**
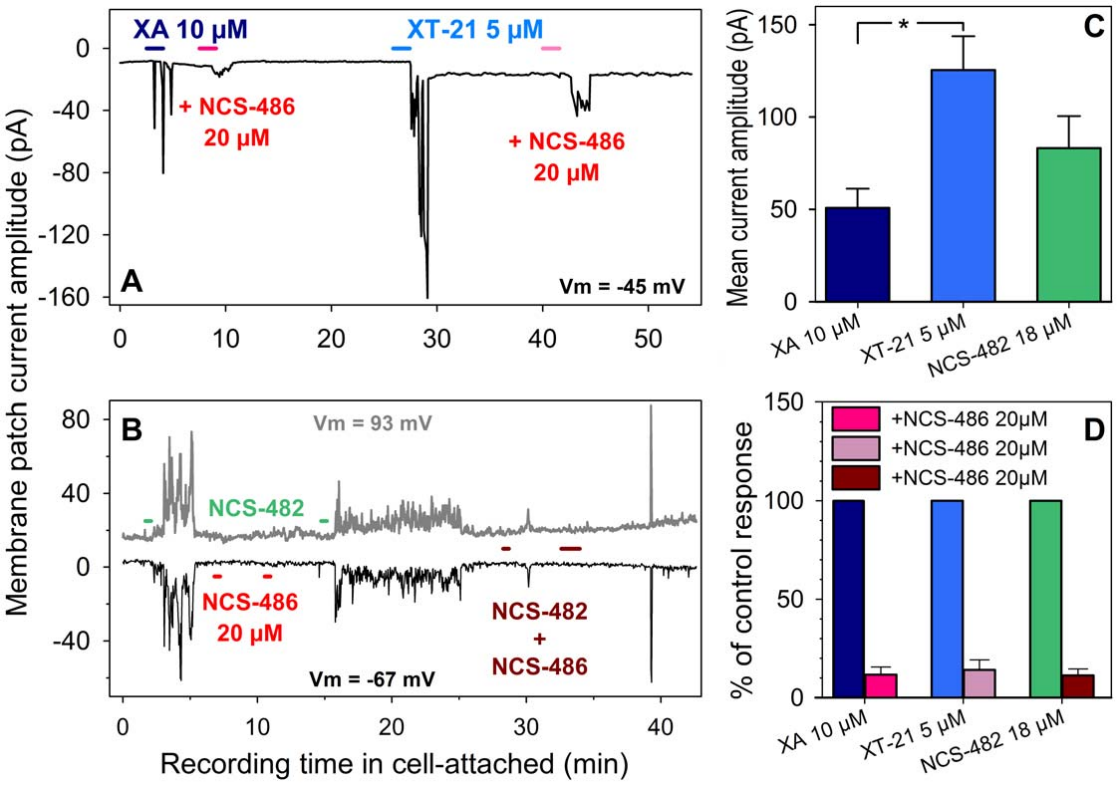
Pharmacological characteristics of XA responses recorded in the cell-attached mode from differentiated NCB-20 cells. **A** and **B**: Agonistic action of XT-21 and NCS-482, and inhibition by NCS-486 of XA, XT-21 and NCS-482 responses. Note the absence of effect of NCS-486 when applied alone (**B**). Horizontal bars represent periods of drug application as indicated. **C**: Statistical data of mean response amplitude (± SEM) obtained with XA, XT-21 and NCS-482 (6, 7 and 4 cells respectively) applied at concentrations as indicated. **D**: Inhibition of the agonist-induced responses by NCS-486 20 µM (12 cells). The mean of agonist responses (control) was set at 100%.

Application of NCS-482 or XT-21 to NCB-20 cells induced similar responses to XA effects, such that at negative membrane potentials an inward current was seen (Figure 8A and 8B). XT-21 was by far the most efficient agonist since 5 µM elicited about 2.5 and 1.5 fold more important response amplitudes than 10 µM XA and 18 µM NCS-482 respectively (Figure 8C).

NCS-486 (20 µM) did not have any effect by itself on NCB-20 cells (Figure 8B; 6 cells tested). However, when co-applied at this same concentration with 10 µM XA, 5 µM XT-21 or 18 µM NCS-482, NCS-486, it strongly inhibited the agonist-induced response such that the current-response was reduced by 88.3±3.8, 86.1±5.0 and 88.7±3.2% respectively (Figure 8D). Like XA, NCS-482 and XT-21 induced responses were inhibited by the same compound (NCS-486), and all these compounds (NCS-482, NCS-486 and XT-21) efficiently displaced [^3^H]-XA binding. These data suggest that the compounds used here competed with the same receptor type and that NCS-486 possesses antagonistic properties at this XA receptor(s).

### Fluo-4 Studies

#### Effects of XA

**Figure 9:**
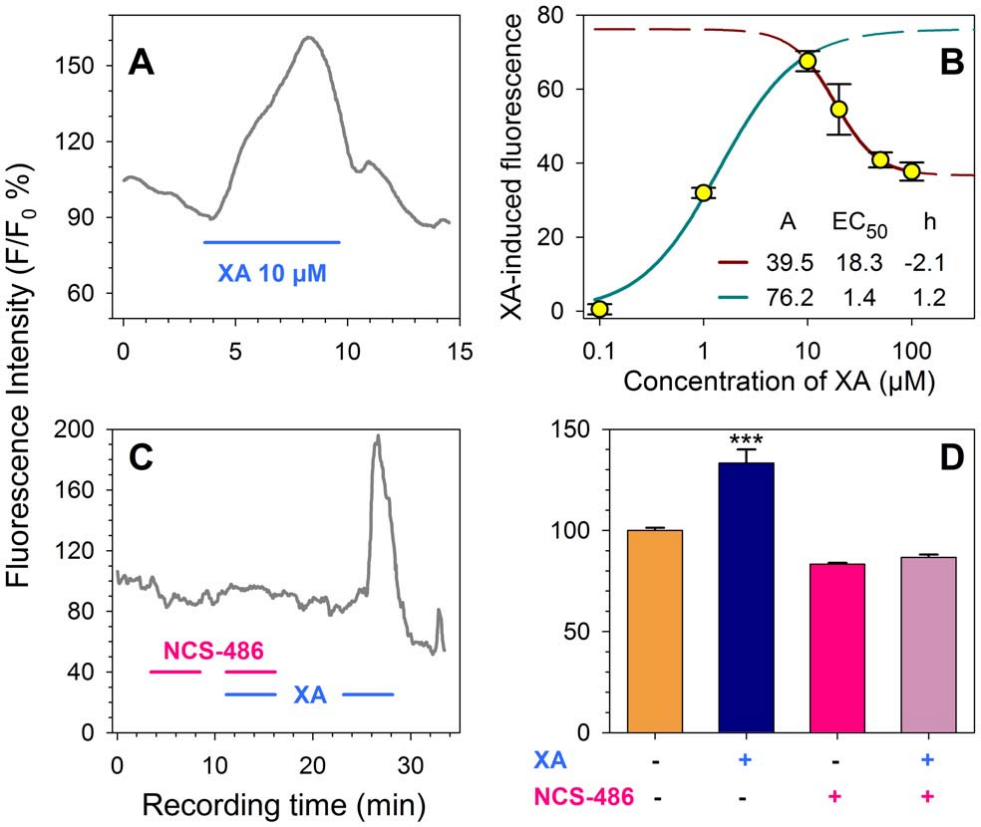
XA-induced intracellular Ca^2+^ increase in differentiated NCB-20 cells. A : Time course of intracellular Ca^2+^ increase detected by fluo-4 probe. The presence of XA 10 µM induced an increase of fluorescence intensity. **B**: Dose-effect of XA-induced response. Data point distribution was biphasic and could be described by activation and partial inactivating phases (green and dark red curves respectively obtained by fitting with the Hill’s equation using optimal parameters as indicated in the figure). **C**: NCS-486 antagonism of the XA-induced cellular calcium response. NCS-486 (100 µM) applied alone or in combination with XA 25 µM did not modify significantly the intracellular Ca^2+^ concentration. After 5 min of washout of the antagonist, XA 25 µM induced a typical increase of fluorescence intensity. **D**: Statistical data of the fluorescence intensity obtained in the presence or absence of XA and/or NCS-486. Results are means±SD obtained from 15 to 50 cells in each condition. Horizontal bar indicates the period of drug application. ***: p<0.001.

Cytosolic Ca^2+^ concentration of NCB-20 cells significantly increased during stimulation with XA. The increase in fluorescence intensity induced by a 6 min application of 10 µM XA is shown in figure 9A and is represented by a large signal starting about 1 min after XA application. The intensity of this signal reached up to 60–80% above basal levels and persisted for about 5 min before returning to basal levels.

The XA-induced response was dose-dependent (Figure 9B). The amplitude of the response increased from 1 to 10 µM XA concentration and reached a mean apparent maximum of about 157±3% (p<0.001). This first phase of the dose-response distribution can be described by the Hill’s equation whose optimal fit to data points gave an EC_50_ value of 1.4 µM that is very close to the EC_50_s obtained for GTP-γ-S (2 µM) and binding (1.3 µM) experiments. However, higher XA concentrations induced a partial decrease of the signal. This decrease was statistically significant and could also be described by the Hill’s equation with an IC_50_ value of 18.3 µM. This partial inactivation could be the result of the well known GPCR arrestin-dependent desensitization mechanism and/or an inactivation linked to an intrinsic cellular calcium regulation.

#### Effect of NCS-486

The effect of the XA receptor antagonist NCS-486 was studied on about 50 cells from several culture batches. Solutions containing 100 µM NCS-486 and/or 25 µM XA were applied to the cells during 5 min intervals as shown in figure 9C. No fluorescence change was detected in the experiments when the antagonist was applied alone (83.9±2.7%) or with XA (86.9±0.7%). However, after a short washout of 5 min, the application of XA induced a significant [Ca^2+^]_i_ increase (134.9±6.5%), confirming the antagonistic properties of NCS-486 (Figure 9C and 9D).

## Discussion

We have recently reported a series of specific properties of XA endogenously present in the rat brain [7]. These results were the first evidence supporting the existence of a specific role of XA in neurotransmission and/or in the regulation of neuronal excitability. To address an important issue in this domain, the present work aimed at demonstrating the existence of specific receptors for XA in the brain. On the basis of kinetic analysis and autoradiographic studies, we showed the existence of XA binding sites with heterogeneous distribution in the rat brain. As it has been shown that XA can bind to mGlu 2/3 receptors, it cannot be excluded that the autoradioraphic images obtained with radioactive XA represent in part the mGlu 2/3 receptors. However, the present images were obtained with the endogenous natural ligand XA while labeling of mGlu 2/3 was carried out with a synthetic rigid analogue of glutamate which possesses high affinity for these receptors [24]. Thus, the autoradiographic images are most probably a superimposition of mGlu 2/3 receptors with the present XA receptor. Some brain regions are almost totally devoid of binding sites. Binding sites appear to be localized on neuronal membranes, but this may not be exclusively the case because the kynurenine pathway is partly present in astrocytes [25] and oligodendrocytes [26]. The affinity of these sites for their endogenous ligand is closely related to the average concentration of XA present in rat brain tissue [7]. Between the classical amino acid neurotransmitters GABA and glutamate, only glutamate had a very weak effect on XA binding suggesting that the contribution of XA binding to glutamate receptor types in our conditions did not exceed about 10%. It could be concluded that the present XA receptor is not glutamate-sensitive and is most probably an original XA receptor.

Furthermore, some structural synthetic analogues of XA also exhibit affinity for XA binding sites, contrasting with the low affinity of most of the kynurenine pathway intermediates. Screening for possible interactions of XA on a collection of 75 other neuronal membrane receptors is also in favor of the existence of XA specific binding sites. Interference with possible glutamate receptors was not tested because of the very low effect of glutamate on XA specific binding. In addition, the electrophysiological responses induced by XA on NCB-20 neurons that show no glutamate-induced stimulation eliminate potential glutamate receptor contributions. Overall, the results reported in this paper lend support to the idea that the XA binding sites are neuronal receptor sites that mediate excitatory responses in brain by involving G proteins. The XA-induced GTP-γ-S binding at optimal MgCl_2_ and GDP concentrations with an EC_50_ close to the Kd of XA for binding sites is an important indication in favor of this assumption. In addition, electrophysiological results using patch-clamp experiments in the cell-attached configuration showed the involvement of cytosolic second messengers in the NCB-20 responses to XA that strongly suggest the involvement of G protein(s).

However, the present XA activated G-protein coupled receptor(s) remains to be identified, together with its possible existence in other tissues, particularly in the human brain. Recently, it has been suggested that XA could be a ligand of class II metabotropic glutamate receptors and based on in vivo recording of rat ventrobasal thalamic neuronal activity the authors suggest that XA could act as an allosteric agonist compound on this class II mGluR [13]. Our autoradiographic results on tissue XA binding cannot exclude cross-binding to mGluR II. However, using differentiated NCB-20 cells which do not express glutamate-inducible intracellular calcium responses [23], we clearly show in the present study that XA is able to induce cytosolic calcium increases. Moreover, the XA-induced functional effects reported here were obtained from cells under continuous superfusion and XA was applied alone, suggesting an agonist rather than an allosteric effect of XA. Furthermore, class II mGluR specific agonists reduce synaptic activity by potassium channel activation [27]. On the contrary, our results revealed a probable excitatory effect of XA through the activation of cationic channels and intracellular calcium increases. All these results strongly suggest that the XA cellular responses we describe here were not mediated by metabotropic glutamate receptors but by distinct receptor(s) which remain to be identified.

Plasma membrane effectors of NCB-20 cells stimulated by XA receptor(s) activation are cationic channels of different selectivity, as revealed by the use of the cell-attached recording mode and NMDG^+^/Mg^2+^ pipette medium. Whether this multiple channel selectivity originates from dynamic changes in channel selectivity 28,29 and/or different channel structures remains to be studied. Whatever the molecular mechanism involved in this channel selectivity or diversity, considering that some XA-activated TRP channels seem to be permeable to divalent cations, they are probably members of the TRPM and/or TRPV channel subfamilies [29]–[31]. Their activation evoked cytosolic Ca^2+^ increase, as evidenced by our experiments using Fluo-4 calcium probes. This cellular Ca^2+^ increase may arise from calcium influx and may include a participation of voltage-dependent Ca^2+^ channels. However, cellular Ca^2+^-store mobilization cannot be excluded. Interestingly, the depolarizing effect resulting from cationic channel activation and the rise in cellular free calcium observed confer an evident excitatory role for XA and suggest that it may facilitate neurotransmitter release.

XA effects were registered at micromolar concentrations which are within the range of the calculated Kd for the binding site. In addition, the effect of synthetic related XA structural analogues suggests a pharmacological specificity of the XA binding/receptor site which is not shared by the natural kynurenine intermediates so far tested. Among the synthetic compounds, NCS-486 appears to display antagonistic properties. These types of synthetic XA related analogues could open new avenues for the synthesis of pharmacological tools and potential therapeutic agents, similar to those developed for kynurenic acid [12].

Finally, the list of human diseases showing abnormal kynurenine pathway metabolism is extensive and diverse [12]. In this context, we propose that the controlled and balanced actions of kynurenic acid and of its related analogue XA participate in the control of inhibitory/excitatory phenomenon in brain, via the crucial regulation of the activity of kynurenine 3-hydroxylase. The deregulation of these dual functions via distinct receptors could be at the base of pathological neurological situations in which the implication of the kynurenine pathway has been suggested.

To summarize, the non-uniform cerebral distribution, kinetic parameters and specific pharmacology of XA binding sites strongly suggest the expression of specific XA receptors in the rat brain, most probably of the GPCR type. These findings suggest a novel function for XA in brain. However, several other methodological approaches are needed to better understand the status of XA among brain kynurenine pathway derivatives, particularly its possible implication in diseases involving tryptophan metabolism oxidative circuits [1], [32]–[34].

## Supporting Information

Figure S1 **Synthesis of the XA analogues NCS-482 and XT-21.**
(TIFF)
